# Impact of SLCO1B1*5 on Flucloxacillin and Co-Amoxiclav–Related Liver Injury

**DOI:** 10.3389/fphar.2022.882962

**Published:** 2022-06-08

**Authors:** Mohammad Alshabeeb, Fadhel A. Alomar, Amjad Khan

**Affiliations:** ^1^ King Abdullah International Medical Research Center (KAIMRC), Riyadh, Saudi Arabia; ^2^ King Saud bin Abdulaziz University for Health Sciences (KSAU-HS), Riyadh, Saudi Arabia; ^3^ Department of Pharmacology and Toxicology, College of Clinical Pharmacy, Imam Abdulrahman Bin Faisal University, Dammam, Saudi Arabia; ^4^ Department of Biological Sciences (Zoology), Faculty of Science, University of Lakki Marwat, Lakki Marwat, Pakistan

**Keywords:** SLCO1B1, DILI, flucloxacillin, co-amoxiclav, liver injury, pharmacogenetics

## Abstract

**Background:** Idiosyncratic drug-induced liver injury (DILI) is a serious uncommon disease that may develop as a result of the intake of certain drugs such as the antimicrobials flucloxacillin and co-amoxiclav. The reported cases showed significant associations between DILI and various human leukocyte (HLA) markers. The solute carrier organic anion transporter 1B1 (SLCO1B1), a non-HLA candidate gene, was previously reported as a risk factor for liver injury induced by rifampin and methimazole. This study presumed that SLCO1B1 may play a general role in the DILI susceptibility and therefore investigated the association of rs4149056 (SLCO1B1*5, T521C) polymorphism with flucloxacillin- and co-amoxiclav–induced liver injury.

**Methodology:** We recruited 155 and 165 DILI cases of white ancestral origin from various European countries but mainly from the United Kingdom owing to flucloxacillin and co-amoxiclav, respectively. Only adult patients (≥18 years) who were diagnosed with liver injury and who showed i) clinical jaundice or bilirubin >2x the upper limit of normal (ULN), ii) alanine aminotransferase (ALT) >5x ULN or iii) alkaline phosphatase (ALP) >2x ULN and bilirubin > ULN were selected. The population reference sample (POPRES), a European control group (*n* = 282), was used in comparison with the investigated cases. TaqMan SNP genotyping custom assay designed by Applied Biosystems was used to genotype both DILI cohorts for SLCO1B1 polymorphism (rs4149056). Allelic discrimination analysis was performed using a step one real-time PCR machine. Genotype differences between cases and controls were examined using Fisher’s exact test. GraphPad Prism version 5.0 was used to determine the *p*-value, odds ratio, and 95% confidence interval. Compliance of the control group with Hardy–Weinberg equilibrium was proven using a web-based calculator available at https://wpcalc.com/en/equilibrium-hardy-weinberg/.

**Results:** A small number of cases failed genotyping in each cohort. Thus, only 149 flucloxacillin and 162 co-amoxiclav DILI cases were analyzed. Genotyping of both DILI cohorts did not show evidence of association with the variant rs4149056 (T521C) (OR = 0.71, 95% CI = 0.46–1.12; *p* = 0.17 for flucloxacillin cases and OR = 0.87, 95% CI = 0.56–1.33; *p* = 0.58 for co-amoxiclav), although slightly lower frequency (22.8%) of positive flucloxacillin cases was noticed than that of POPRES controls (29.4%).

**Conclusion:** Carriage of the examined allele SLCO1B1*5 is not considered a risk factor for flucloxacillin DILI or co-amoxiclav DILI as presumed. Testing a different allele (SLCO1B1*1B) and another family member gene (SLCO1B3) may still be needed to provide a clearer role of SLCO1B drug transporters in DILI development–related to the chosen antimicrobials.

## Introduction

Drug-induced liver injury (DILI) is an uncommon but serious adverse reaction that may develop upon exposure to several drugs (Andrade et al., 2019). Antibiotics are considered the most commonly implicated drug category in idiosyncratic DILI development (Katarey and Verma, 2016; Björnsson, 2017). Flucloxacillin and co-amoxiclav are well-tolerated beta-lactam penicillins and widely used in clinical practice with an acceptable safety profile; however, they have been reported to cause DILI (Donati et al., 2017; Lindh et al., 2018; Teixeira et al., 2020).

Despite the increased number of studies to identify genetic risk factors contributed to DILI development, the exact genetic profile for propensity to DILI is not fully understood, with some important exceptions. Several reports demonstrated a strong influence of human leukocyte antigen (HLA) genes on DILI susceptibility due to flucloxacillin and co-amoxiclav, though the overall effects are small (Donaldson et al., 2008; Daly et al., 2009). Thus, it is likely that the non-HLA markers may also contribute. Drug transporters play an important role in drug absorption, extent of tissue penetration, and excretion. The transporter’s altered function as a result of genetic polymorphism may lead to idiosyncratic toxicity (DeGorter et al., 2012). An example of a drug uptake transporter is the organic anion transporting polypeptide 1B1 (OATP1B1), encoded by solute carrier organic anion-transporting polypeptide 1B1 (SLCO1B1) (Niemi et al., 2011). This gene is exclusively expressed on the liver sinusoidal membrane and functions as a mediator for uptake and clearance of bilirubin, bile acids, and xenobiotic compounds such as statins, penicillins (Yamaguchi et al., 2011), and certain cancer agents, including irinotecan and methotrexate, from blood stream by transporting them into the liver (Niemi et al., 2011). The transporting functions of OATP1B1 can be significantly affected by selected SLCO1B1 variants (Kasai and Ikeda, 2011; Jones et al., 2012; Nies et al., 2013). The variant rs4149056 in SLCO1B1 (SLCO1B1*5) is a non-synonymous mutation that results in an amino acid change from valine (T allele) to alanine (C allele) at codon 174. SLCO1B1*5 was found to be a strong predictor to statin-induced myopathy (Link et al., 2008; Carr et al., 2013). It has also shown to be a risk factor for several other types of adverse drug reactions such as anemia and GI toxicity associated with the administration of regorafenib and methotrexate, respectively (Treviño et al., 2009; Maeda et al., 2017). Moreover, a meta-analysis study revealed a significant influence of SLCO1B1*5 on elevated serum levels of bilirubin and gallstone composition (Johnson et al., 2009; Buch et al., 2010; Srivastava et al., 2011). In a knockout mouse model, animals carrying a mutated SLCO1B2 gene, the closest orthologic gene of the human SLCO1B1 and SLCO1B3, showed lower liver uptake of certain tested drugs (pravastatin, lovastatin, and rifampicin) compared to that of wild-type controls (DeGorter et al., 2012). These results are consistent with previous human studies that emphasized lower hepatic uptake of a variety of statins and rifampicin in patients carrying SLCO1B1 variants (Li et al., 2012; Meyer zu Schwabedissen et al., 2015; Wu et al., 2018). Accumulation of xenobiotics in plasma causes further inhibition in hepatic uptake and excretion of bile acids, which underlies the formation of gallstones and development of liver injury (Kemp et al., 2005; Li et al., 2012). Although both SLCO1B1 and SLCO1B3 transporters may contribute to hepatic penicillin uptake (Yamaguchi et al., 2011), data on functional significance of polymorphisms in SLCO1B3 transporter are limited. Therefore, this study has focused on SLCO1B1 as a candidate gene for flucloxacillin- and co-amoxiclav–related DILI focusing on SLCO1B1*5 (rs4149056, T521C).

## Materials and Methods

### DILI Cases

A total of 155 flucloxacillin DILI and 165 co-amoxiclav DILI cases were recruited based on the inclusion criteria described below. All cases were white European patients recruited from various European countries including Netherlands, Iceland, Sweden, but the majority were recruited from the United Kingdom. The clinical and biochemical features of the flucloxacillin cases are provided in Table 1, whereas the patient characteristics of co-amoxiclav DILI cases were described in a previous project (Alshabeeb et al., 2020). All data were extracted from two previous studies DILIGEN and iDILIC, which were described in detail by Donaldson et al. (2010), Lucena et al. (2011), Nicoletti et al. (2017), and Cirulli et al. (2019).

**TABLE 1:** Clinical and biochemical variables in DILI patients exposed to flucloxacillin.

Demographic data of flucloxacillin DILI participants (*n* = 155)	Descriptive value (%)
Sex (F/M)	108 (69.7)/47 (30.3)
Age at onset (years, mean ± SD)	62.6 ± 13.4
Time to onset (days, mean ± SD)	23.5 ± 17.8
Duration on drug (days, mean ± SD)	10.3 ± 6.2
Pattern of liver injury	
Cholestatic	95 (61.3)
Hepatocellular	13 (8.4)
Mixed	47 (30.3)
RUCAM scoring	
3–5 (possible)	17 (11.0)
6–8 (probable)	63 (40.6)
>8 (highly probable)	75 (48.4)
Peak bilirubin (µmol/l)	264.3 ± 227.1
Peak ALT (U/l)	400.4 ± 253.9
Peak ALP (U/l)	569.6 ± 668.5
Severity of DILI	
Mild	11 (7.1)
Moderate	139 (89.7)
Severe	3 (1.9)
Very severe	2 (1.3)
Cases with jaundice	144 (92.9)
Time taken for ALT/ALP to decrease to ≥50% after drug discontinuation (days)	68.4 ± 74.3
Cases positive for HLA:B*57:01 (%)	131 (84.5)

### Inclusion Criteria and Patient Eligibility

Patients (≥18 years) need to fulfill one of the following three diagnostic criteria to be recruited: 1) clinical jaundice or bilirubin >2x the upper limit of normal (ULN), 2) alanine aminotransferase (ALT) >5x ULN, or 3) alkaline phosphatase (ALP) >2x ULN and bilirubin > ULN. Informed consents of all patients were signed and drug causality by either flucloxacillin or co-amoxiclav was adjudicated by two expert hepatologists (Professor Guruprasad Aithal and Dr Einar Bjornsson) based on the international consensus criteria (RUCAM scoring) (Bénichou, 1990; Aithal et al., 2011).

### Control Samples

The population reference sample, POPRES (*n* = 282) was used as the control group. The United Kingdom individuals from this cohort are described by Nelson et al. (2008). The POPRES group is a resource for population, disease, and pharmacological genetics research that has been shown to be a good genetic match for the flucloxacillin and co-amoxiclav cases included in the GWAS for DILI due to these drugs (Daly et al., 2009; Lucena et al., 2011). The population controls used were not necessarily drug treated since drug histories on the population controls were not available. However, it is widely accepted to use drug non-exposed controls in genetic studies involving comparisons with very rare diseases affecting <1% such as DILI due to the very low likelihood that controls would ever develop the disease (Nelson et al., 2009). Also, recruitment of controls for the study who had been prescribed flucloxacillin and/or co-amoxiclav and following them up would be expensive and time-consuming and was not feasible, given the limited resources and time. Cases and controls were not matched for age and sex as the demographic data for the used control group are not available.

### TaqMan Assay

TaqMan SNP genotyping custom assay was used to genotype both DILI cohorts for SLCO1B1 polymorphism (rs4149056). It was designed by Applied Biosystems and delivered as 20X single-tube mixtures (188 µl) of forward and reverse primers (900 µM) in addition to two reporter probes (200 µM). The 5′ end of each probe is linked to different fluorescent allele–specific dyes; a fluorescein amidite (FAM) is allele 2 (C)–specific, while VIC is the reporter for allele 1 (T). The 2X TaqMan universal PCR master mix (Applied Biosystems) was used which contains AmpliTaq Gold^®^ DNA polymerase, dNTP, and passive internal reference based on proprietary ROX dye.

To prepare the reaction mix to amplify 48 samples in 48-well plates, 15 µl of 20X working stock of SNP genotyping assay was added to 285 µl of 2X universal master mix and diluted with 200 µl of distilled water. After vortexing, 10 µl of the mixture was transferred into each well of the 48 reaction plates. Next, 20 ng of wet genomic DNA was added. The plate was sealed and inserted into the one-step Applied Biosystems real-time PCR machine. The PCR temperature was maintained on hold for 10 min at 95°C, then reduced to 92°C for 15 s (denaturation), and further reduced in annealing and extension stages to 60°C for 1 min each for 40 cycles.

## Statistical Analysis

Differences between tested cases and control genotypes were examined using Fisher’s exact test. *p*-value, odds ratio, and 95% confidence interval were calculated using GraphPad Prism version 5.0. Compliance with Hardy–Weinberg equilibrium was calculated for the control group to confirm that they met standard quality criteria using a web-based calculator available at https://wpcalc.com/en/equilibrium-hardy-weinberg/.

## Genotyping Results

Six of flucloxacillin cases and three of co-amoxicillin cases failed genotyping; therefore, the remaining analyzed samples were 149 and 162, respectively. Analysis of TaqMan genotyping results of both co-amoxiclav and flucloxacillin DILI cohorts did not show evidence of association with the tested groups (OR = 0.71, 95% CI = 0.46–1.12; *p* = 0.17) for flucloxacillin cases and OR = 0.87, 95% CI = 0.56–1.33; *p* = 0.58 for co-amoxiclav, Table 2). Despite the observed slightly lower frequency of positive flucloxacillin cases (22.8%) than that of POPRES controls (29.4%), this difference was not significant. Subgroup analysis of flucloxacillin cases negative (*n* = 24) and positive for rs2395029 (*n* = 125), a tag SNP in full linkage disequilibrium (LD) with HLA-B*57:01, which was described as major determinant of flucloxacillin DILI (Daly et al., 2009), was also performed but failed to detect a role for SLCO1B1 in flucloxacillin DILI susceptibility (for negative cases: OR = 0.48, 95% CI = 0.16–1.45; *p* = 0.24, for positive cases: OR = 0.76, 95% CI = 0.47–1.23; *p* = 0.28). In addition, we investigated the contribution of SLCO1B1 to DILI in the cases with cholestatic or mixed DILI within the flucloxacillin group, but there was no effect observed (OR = 0.90, 95% CI = 0.57–1.41; *p* = 0.64).

**TABLE 2:** SLCO1B1*5 (rs4149056) genotyping results in both DILI cohorts compared to POPRES community controls.

Genotype	TT (%)	CT (%)	CC (%)	*p*-value	Odds ratio	95% CI
Flucloxacillin cases (*n* = 149)	115 (77.2)	31 (20.8)	3 (2.0)	0.17	0.71	0.46–1.12
Flucloxacillin cases positive for rs2395029 (tag SNP for HLA-B*57:01) (*n* = 125)	95 (76)	27 (21.6)	3 (2.4)	0.28	0.76	0.47–1.23
Flucloxacillin cases with cholestatic or mixed phenotypes^*^(*n* = 136)	104 (76.5)	29 (21.3)	3 (2.2)	0.64	0.90	0.57–1.41
Co-amoxicillin cases (*n* = 162)	119 (73.4)	40 (24.7)	3 (1.9)	0.58	0.87	0.56–1.33
POPRES controls (*n* = 282)	199 (70.6)	74 (26.2)	9 (3.2)			

^*^Cases with mixed phenotypes are those which show both cholestatic and hepatocellular clinical manifestations.

## Discussion

Drug-related adverse hepatic reactions are thought to evolve either due to a direct exposure of hepatocytes to toxic parent drugs or their metabolites (non-immune pathway) or as a result of inflammatory mediator activation (immune-mediated), though the exact mechanisms by which drugs enhance liver toxicity is not clear yet (Kaplowitz and DeLeve, 2013). The immune-mediated DILI is commonly accompanied by the classical allergic reactions including eosinophilia, fever, and rash, although absence of such reactions does not necessarily exclude immune response contribution to DILI as in the case of drug-induced autoimmunity (Uetrecht, 2008). Numerous environmental and genetic factors have been suggested to play a role in the induction of metabolic-related DILI. In patients with SLCO1B1 variants, which inhibit hepatic uptake of drugs and bile acids, the elevated bile salts are predicted to trigger antimicrobial-induced cholestasis and liver inflammation (Li et al., 2012).

Some genetic factors may play a general role for DILI susceptibility rather than being drug-specific (Daly and Day, 2009). The previous reported findings suggested SLCO1B1*15, a haplotype that includes SLCO1B1*5 and SLCO1B1*1B (rs2306283 and A388G), as a risk factor for drug-induced liver injury (DILI) due to rifampin (Li et al., 2012) and methimazole (Jin et al., 2019). In addition, individuals positive for SLCO1B1*15 showed higher risk to develop liver damage when exposed to high levels of aflatoxin B1 (AFB1), a compound commonly found in contaminated food (Yang et al., 2017). Moreover, hyperlipidemic patients who are positive for SLCO1B1*15 showed the highest abnormal levels of liver enzymes (ALT and AST) (Wu et al., 2018). Based on the documented associations of SLCO1B1 with DILI, this study intended for the first time to investigate SLCO1B1*5 (rs4149056) as a potential candidate gene to DILI development related to the antimicrobials flucloxacillin and co-amoxiclav. The minimum allele frequency (MAF) was carried by 16.3% of the POPRES white European population control group used in this study, which is consistent to the frequency (17%) reported in a larger study on 1,105 healthy volunteers from 18 different European countries (Mizzi et al., 2016), though inter-ethnic differences related to the tested SLCO1B1 allelic variant were noted among these countries, in particular in Polish, Cypriot, and Lithuanian (MAF = 35%, 7%, and 38%, respectively). The reported allele frequency among South African and Saudi Arabian is higher than the average frequency seen in Europeans of white origin (MAF = 22% and 27% vs. 17%) (Mizzi et al., 2016).

Our findings showed no significant associations between the examined variant and DILI in relation to either flucloxacillin or co-amoxiclav. The most common patterns of DILI are a cholestatic phenotype due to bile duct obstruction and disturbances in bile secretion, a hepatocellular phenotype that is characterized by the elevation of liver enzymes, particularly ALT, or a mixed presentation (Hoofnagle and Björnsson, 2019). The previous reports on flucloxacillin DILI cases emphasized that a stronger genetic association can be detected in patients with cholestatic or mixed (cholestatic and hepatocellular) phenotypes (Russmann et al., 2005; Daly et al., 2009). Subgroup analyses in this study which focused on patients with cholestatic and mixed injury features also did not show any genotype–phenotype associations. Restricting the analysis for the cases positive for the HLA-B*57:01 marker, previously identified as the most important allele that impacted flucloxacillin hepatotoxicity (Daly et al., 2009), was attempted to investigate if a synergy exists between HLA-B*57:01 and SLCO1B1*5. Our results were negative, and the influence of the HLA variant had been suppressed when SLCO1B1*5 was considered.

The SLCO1B1*1B was previously reported as the major determinant of the expression of the OATP1B1 receptor in the liver (Nies et al., 2013); therefore, it is necessary to genotype both alleles (SLCO1B1*5 and SLCO1B1*1B) to determine the impact of the SLCO1B1 haplotype (SLCO1B1*15) on flucloxacillin and co-amoxiclav DILI cases.

Interestingly, in view of the apparently more important role for SLCO1B3 than that of SLCO1B1 in the transport of penicillins and related compounds (Yamaguchi et al., 2011), genotyping for SNPs in this second transporter gene would be worthwhile in the future. SLCO1B1 and 1B3 are both located on chromosome 12p12 and there is linkage disequilibrium between polymorphisms in both genes. In particular, a recent report has shown that the SNP genotypes of rs4149056 (Val174Ala) in SLCO1B1 are in linkage disequilibrium with several SNPs in SLCO1B3 (Nies et al., 2013). Some of these linked SNPs in SLCO1B3 are non-synonymous and associated with impaired transport of the immunosuppressant tacrolimus (Boivin et al., 2013). Common SLCO1B3 variants were found to be risk factors for hyperbilirubinemia in adults (Sanna et al., 2009) and neonates (Alencastro de Azevedo et al., 2012). Hence, studying this gene directly in penicillin-related DILI would still be appropriate.

In conclusion, this study indicated that the previous reported DILI associations with SLCO1B1 are either drug-specific (e.g., rifampin and methimazole) or related to a haplotype, including both SLCO1B1*5 and SLCO1B1*1B, rather than associated to a single marker only. Thus, no associations were detected between neither flucloxacillin DILI nor co-amoxiclav DILI with the tested allele (SLCO1B1*5).

## Data Availability

The original contributions presented in the study are included in the article/Supplementary Material; further inquiries can be directed to the corresponding author.

## References

[B1] Aithal G. P. Watkins P. B. Andrade R. J. Larrey D. Molokhia M. Takikawa H. (2011). Case Definition and Phenotype Standardization in Drug-Induced Liver Injury. Clin. Pharmacol. Ther. 89 (6), 806–815. 10.1038/clpt.2011.58 21544079

[B2] Alencastro de Azevedo L. Reverbel da Silveira T. Carvalho C. G. Martins de Castro S. Giugliani R. Matte U. (2012). UGT1A1, SLCO1B1, and SLCO1B3 Polymorphisms vs. Neonatal Hyperbilirubinemia: Is There an Association? Pediatr. Res. 72 (2), 169–173. 10.1038/pr.2012.60 22580719

[B3] Alshabeeb M. A. Aithal G. P. Daly A. K. (2020). Investigation of Oxidative Stress-Related Candidate Genes as Risk Factors for Drug-Induced Liver Injury Due to Co-amoxiclav. DNA Cell Biol. 39 (3), 349–354. 10.1089/dna.2019.4982 31905014

[B4] Andrade R. J. Chalasani N. Björnsson E. S. Suzuki A. Kullak-Ublick G. A. Watkins P. B. (2019). Drug-induced Liver Injury. Nat. Rev. Dis. Prim. 5 (1), 58. 10.1038/s41572-019-0105-0 31439850

[B5] Bénichou C. (1990). Criteria of Drug-Induced Liver Disorders. Report of an International Consensus Meeting. J. Hepatol. 11 (2), 272–276. 10.1016/0168-8278(90)90124-a 2254635

[B6] Björnsson E. S. (2017). Drug-induced Liver Injury Due to Antibiotics. Scand. J. gastroenterology 52 (6-7), 617–623. 10.1080/00365521.2017.1291719 28276834

[B7] Boivin A. A. Cardinal H. Barama A. Naud J. Pichette V. Hébert M. J. (2013). Influence of SLCO1B3 Genetic Variations on Tacrolimus Pharmacokinetics in Renal Transplant Recipients. Drug Metab. Pharmacokinet. 28 (3), 274–277. 10.2133/dmpk.dmpk-12-sh-093 23149872

[B8] Buch S. Schafmayer C. Völzke H. Seeger M. Miquel J. F. Sookoian S. C. (2010). Loci from a Genome-wide Analysis of Bilirubin Levels Are Associated with Gallstone Risk and Composition. Gastroenterology 139 (6), 1942–e2. 10.1053/j.gastro.2010.09.003 20837016

[B9] Carr D. F. O'meara H. Jorgensen A. L. Campbell J. Hobbs M. McCann G. (2013). SLCO1B1 Genetic Variant Associated with Statin-Induced Myopathy: a Proof-Of-Concept Study Using the Clinical Practice Research Datalink. Clin. Pharmacol. Ther. 94 (6), 695–701. 10.1038/clpt.2013.161 23942138 PMC3831180

[B10] Cirulli E. T. Nicoletti P. Abramson K. Andrade R. J. Bjornsson E. S. Chalasani N. (2019). A Missense Variant in PTPN22 Is a Risk Factor for Drug-Induced Liver Injury. Gastroenterology 156 (6), 1707–e2. 10.1053/j.gastro.2019.01.034 30664875 PMC6511989

[B11] Daly A. K. Day C. P. (2009). Genetic Association Studies in Drug-Induced Liver Injury. Semin. Liver Dis. 29 (4), 400–411. 10.1055/s-0029-1240009 19826974

[B12] Daly A. K. Donaldson P. T. Bhatnagar P. Shen Y. Pe'er I. Floratos A. (2009). HLA-B*5701 Genotype Is a Major Determinant of Drug-Induced Liver Injury Due to Flucloxacillin. Nat. Genet. 41 (7), 816–819. 10.1038/ng.379 19483685

[B13] DeGorter M. K. Xia C. Q. Yang J. J. Kim R. B. (2012). Drug Transporters in Drug Efficacy and Toxicity. Annu. Rev. Pharmacol. Toxicol. 52, 249–273. 10.1146/annurev-pharmtox-010611-134529 21942630

[B14] Donaldson P. Bhatnagar P. Graham P. Henderson J. Leathart J. Pirmohamed M. (2008). Susceptibility to Drug-Induced Liver Injury Determined by HLA Class II Genotype. Hepatology 48 (4). 10.1002/hep.22641

[B15] Donaldson P. T. Daly A. K. Henderson J. Graham J. Pirmohamed M. Bernal W. (2010). Human Leucocyte Antigen Class II Genotype in Susceptibility and Resistance to Co-amoxiclav-induced Liver Injury. J. Hepatol. 53 (6), 1049–1053. 10.1016/j.jhep.2010.05.033 20800921

[B16] Donati M. Motola D. Leone R. Moretti U. Stoppa G. Arzenton E. (2017). Liver Injury Due to Amoxicillin vs. Amoxicillin/clavulanate: A Subgroup Analysis of a Drug-Induced Liver Injury Case-Control Study in Italy. J. Hepatol. Gastrointest. Disord. 3 (01). 10.4172/2475-3181.1000143

[B17] Hoofnagle J. H. Björnsson E. S. (2019). Drug-Induced Liver Injury - Types and Phenotypes. N. Engl. J. Med. 381 (3), 264–273. 10.1056/nejmra1816149 31314970

[B18] Jin S. Li X. Fan Y. Fan X. Dai Y. Lin H. (2019). Association between Genetic Polymorphisms of SLCO1B1 and Susceptibility to Methimazole-Induced Liver Injury. Basic Clin. Pharmacol. Toxicol. 125 (6), 508–517. 10.1111/bcpt.13284 31240859

[B19] Johnson A. D. Kavousi M. Smith A. V. Chen M. H. Dehghan A. Aspelund T. (2009). Genome-wide Association Meta-Analysis for Total Serum Bilirubin Levels. Hum. Mol. Genet. 18 (14), 2700–2710. 10.1093/hmg/ddp202 19414484 PMC2701336

[B20] Jones M. B. Nasirikenari M. Lugade A. A. Thanavala Y. Lau J. T. (2012). Anti-inflammatory IgG Production Requires Functional P1 Promoter in β-galactoside α2,6-sialyltransferase 1 (ST6Gal-1) Gene. J. Biol. Chem. 287 (19), 15365–15370. 10.1074/jbc.M112.345710 22427662 PMC3346113

[B21] Kaplowitz N. DeLeve L. (2013). Drug-induced Liver Disease. 3 rd ed. USA: Elsevier.

[B22] Kasai S. Ikeda K. (2011). Pharmacogenomics of the Human µ-Opioid Receptor. Pharmacogenomics 12 (9), 1305–1320. 10.2217/pgs.11.68 21919606

[B23] Katarey D. Verma S. (2016). Drug-induced Liver Injury. Clin. Med. (Lond) 16 (Suppl. 6), s104. 10.7861/clinmedicine.16-6-s104 27956449 PMC6329561

[B24] Kemp D. C. Zamek-Gliszczynski M. J. Brouwer K. L. (2005). Xenobiotics Inhibit Hepatic Uptake and Biliary Excretion of Taurocholate in Rat Hepatocytes. Toxicol. Sci. 83 (2), 207–214. 10.1093/toxsci/kfi020 15509663

[B25] Li L. M. Chen L. Deng G. H. Tan W. T. Dan Y. J. Wang R. Q. (2012). SLCO1B1 *15 Haplotype Is Associated with Rifampin-Induced Liver Injury. Mol. Med. Rep. 6 (1), 75–82. 10.3892/mmr.2012.900 22562052

[B26] Lindh M. Hallberg P. Yue Q. Y. Wadelius M. (2018). Clinical Factors Predicting Drug-Induced Liver Injury Due to Flucloxacillin. Drug Healthc. Patient Saf. 10, 95–101. 10.2147/DHPS.S178394 30538582 PMC6254585

[B27] Link E. Link E. Parish S. Armitage J. Bowman L. Heath S. (2008). SLCO1B1 Variants and Statin-Induced Myopathy-Aa Genomewide Study. N. Engl. J. Med. 359 (8), 789–799. 10.1056/NEJMoa0801936 18650507

[B28] Lucena M. I. Molokhia M. Shen Y. Urban T. J. Aithal G. P. Andrade R. J. (2011). Susceptibility to Amoxicillin-Clavulanate-Induced Liver Injury Is Influenced by Multiple HLA Class I and II Alleles. Gastroenterology 141 (1), 338–347. 10.1053/j.gastro.2011.04.001 21570397 PMC3129430

[B29] Maeda A. Ando H. Ura T. Komori A. Hasegawa A. Taniguchi H. (2017). Association between ABCG2 and SLCO1B1 polymorphisms and adverse drug reactions to regorafenib: a preliminary study. Int. J. Clin. Pharmacol. Ther. 55 (5), 409–415. 10.5414/CP202788 28157071

[B30] Meyer zu Schwabedissen H. E. Albers M. Baumeister S. E. Rimmbach C. Nauck M. Wallaschofski H. (2015). Function-impairing Polymorphisms of the Hepatic Uptake Transporter SLCO1B1 Modify the Therapeutic Efficacy of Statins in a Population-Based Cohort. Pharmacogenet Genomics 25 (1), 8–18. 10.1097/FPC.0000000000000098 25379722

[B31] Mizzi C. Dalabira E. Kumuthini J. Dzimiri N. Balogh I. Başak N. (2016). A European Spectrum of Pharmacogenomic Biomarkers: Implications for Clinical Pharmacogenomics. PloS one 11 (9), e0162866. 10.1371/journal.pone.0162866 27636550 PMC5026342

[B32] Nelson M. R. Bacanu S. A. Mosteller M. Li L. Bowman C. E. Roses A. D. (2009). Genome-wide Approaches to Identify Pharmacogenetic Contributions to Adverse Drug Reactions. Pharmacogenomics J. 9 (1), 23–33. 10.1038/tpj.2008.4 18301416

[B33] Nelson M. R. Bryc K. King K. S. Indap A. Boyko A. R. Novembre J. (2008). The Population Reference Sample, POPRES: a Resource for Population, Disease, and Pharmacological Genetics Research. Am. J. Hum. Genet. 83 (3), 347–358. 10.1016/j.ajhg.2008.08.005 18760391 PMC2556436

[B34] Nicoletti P. Aithal G. P. Bjornsson E. S. Andrade R. J. Sawle A. Arrese M. (2017). Association of Liver Injury from Specific Drugs, or Groups of Drugs, with Polymorphisms in HLA and Other Genes in a Genome-wide Association Study. Gastroenterology 152 (5), 1078–1089. 10.1053/j.gastro.2016.12.016 28043905 PMC5367948

[B35] Niemi M. Pasanen M. K. Neuvonen P. J. (2011). Organic Anion Transporting Polypeptide 1B1: a Genetically Polymorphic Transporter of Major Importance for Hepatic Drug Uptake. Pharmacol. Rev. 63 (1), 157–181. 10.1124/pr.110.002857 21245207

[B36] Nies A. T. Niemi M. Burk O. Winter S. Zanger U. M. Stieger B. (2013). Genetics Is a Major Determinant of Expression of the Human Hepatic Uptake Transporter OATP1B1, but Not of OATP1B3 and OATP2B1. Genome Med. 5 (1), 1–11. 10.1186/gm405 23311897 PMC3706890

[B37] Russmann S. Kaye J. A. Jick S. S. Jick H. (2005). Risk of Cholestatic Liver Disease Associated with Flucloxacillin and Flucloxacillin Prescribing Habits in the UK: Cohort Study Using Data from the UK General Practice Research Database. Br. J. Clin. Pharmacol. 60 (1), 76–82. 10.1111/j.1365-2125.2005.02370.x 15963097 PMC1884907

[B38] Sanna S. Busonero F. Maschio A. McArdle P. F. Usala G. Dei M. (2009). Common Variants in the SLCO1B3 Locus Are Associated with Bilirubin Levels and Unconjugated Hyperbilirubinemia. Hum. Mol. Genet. 18 (14), 2711–2718. 10.1093/hmg/ddp203 19419973 PMC2701337

[B39] Srivastava A. Srivastava A. Srivastava N. Choudhuri G. Mittal B. (2011). Organic Anion Transporter 1B1 (SLCO1B1) Polymorphism and Gallstone Formation: High Incidence of Exon4 CA Genotype in Female Patients in North India. Hepatol. Res. 41 (1), 71–78. 10.1111/j.1872-034X.2010.00736.x 20973885

[B40] Teixeira M. Macedo S. Batista T. Martins S. Correia A. Matos L. C. (2020). Flucloxacillin-Induced Hepatotoxicity - Association with HLA-B*5701. Rev. Assoc. Med. Bras. (1992) 66, 12–17. 10.1590/1806-9282.66.1.12 32130375

[B41] Treviño L. R. Shimasaki N. Yang W. Panetta J. C. Cheng C. Pei D. (2009). Germline Genetic Variation in an Organic Anion Transporter Polypeptide Associated with Methotrexate Pharmacokinetics and Clinical Effects. J. Clin. Oncol. 27 (35), 5972. 10.1200/JCO.2008.20.4156 19901119 PMC2793040

[B42] Uetrecht J. (2008). Idiosyncratic Drug Reactions: Past, Present, and Future. Chem. Res. Toxicol. 21 (1), 84–92. 10.1021/tx700186p 18052104

[B43] Wu X. Gong C. Weinstock J. Cheng J. Hu S. Venners S. A. (2018). Associations of the SLCO1B1 Polymorphisms with Hepatic Function, Baseline Lipid Levels, and Lipid-Lowering Response to Simvastatin in Patients with Hyperlipidemia. Clin. Appl. Thromb. Hemost. 24 (Suppl. 9), 240S–247S. 10.1177/1076029618805863 30336686 PMC6714829

[B44] Yamaguchi H. Takeuchi T. Okada M. Kobayashi M. Unno M. Abe T. (2011). Screening of Antibiotics that Interact with Organic Anion-Transporting Polypeptides 1B1 and 1B3 Using Fluorescent Probes. Biol. Pharm. Bull. 34 (3), 389–395. 10.1248/bpb.34.389 21372390

[B45] Yang X. Liu W. Lin H. Zeng H. Zhang R. Pu C. (2017). Interaction Effects of AFB1 and MC-LR Co-exposure with Polymorphism of Metabolic Genes on Liver Damage: Focusing on SLCO1B1 and GSTP1. Sci. Rep. 7 (1), 16164–16210. 10.1038/s41598-017-16432-z 29170472 PMC5700940

